# Applying PICRUSt and 16S rRNA functional characterisation to predicting co-digestion strategies of various animal manures for biogas production

**DOI:** 10.1038/s41598-021-99389-4

**Published:** 2021-10-07

**Authors:** Grace N. Ijoma, Rosina Nkuna, Asheal Mutungwazi, Charles Rashama, Tonderayi S. Matambo

**Affiliations:** Institute for the Development of Energy for African Sustainability, University of South Africa, Roodepoort, 1709 South Africa

**Keywords:** Biochemistry, Computational biology and bioinformatics

## Abstract

An estimated 25 million tons of animal manure is produced globally every year, causing considerable impact to the environment. These impacts can be managed through the use of anaerobic digestion (AD) This process achieves waste degradation through enzymatic activity, the efficiency of the AD process is directly related to microorganisms that produce these enzymes. Biomethane potential (BMP) assays remain the standard theoretical framework to pre-determine biogas yield and have been used to determine the feasibility of substrates or their combination for biogas production. However, an integrated approach that combines substrate choice and co-digestion would provide an improvement to the current predictive models. PICRUSt (Phylogenetic Investigation of Communities by Reconstruction of Unobserved States) addresses the limitations of assays in this regard. In this paper, the biochemical functions of horse, cow, and pig manures are predicted. A total of 135 predicted KEGG Orthologies (KOs) showed amino acids, carbohydrate, energy, lipid, and xenobiotic metabolisms in all the samples. Linear discriminant analysis (LDA) combined with the effect size measurements (LEfSe), showed that fructose, mannose, amino acid and nucleotide sugar, phosphotransferase (PST) as well as starch and sucrose metabolisms were significantly higher in horse manure samples. 36 of the KOs were related to the acidogenesis and/or acetogenesis AD stages. Extended bar plots showed that 11 significant predictions were observed for horse-cow, while 5 were predicted for horse-pig and for cow-pig manures. Based on these predictions, the AD process can be enhanced through co-digestion strategies that takes into account the predicted metabolic contributions of the manure samples. The results supported the BMP calculations for the samples in this study. Biogas yields can be improved if this combined approach is employed in routine analysis before co-digesting different substrates.

## Introduction

Agricultural processes as well as other industrial activities are synonymous with the generation of significant waste. The uncontrolled degradation of these wastes is harmful to the local ecological system^1^. The agricultural animal husbandry sector further generates considerable quantities of animal waste^2^. There is evidence that the uncontrolled emission of biomethane and other greenhouse gases^3^ contribute significantly to the environmental impact of agricultural activities. Moreover, activities in this sector also have considerable demand for potable water^4^ and require vast areas of land for grazing and waste disposal^5^. These wastes may sometimes become repositories for zoonotic infections^6^. Additionally, the high concentrations of nitrogen and phosphorus in the wastes tend to leach into waterbodies exacerbating the environmental consequences through eutrophication and algal blooms, rendering the surrounding waterbodies unfit for domestic use^7, 8^. Yet the demand for meat products and the economic viability of the meat industries implies that commercial livestock rearing, and meat production will remain aspects of our day-to-day living. Thus, realistic and feasible solutions to waste management should integrate environmental mitigation strategies that routinely utilise the organic wastes generated from the processes to address the waste-to-energy conundrum^9–14^. This is especially essential considering that globally, pollution from animal manure is estimated at 25 million tons, annually^15^. The degradation products of animal waste have potential in the energy market, a potential which is currently not being fully harnessed.

The anaerobic digestion (AD) of animal manure to produce biogas has the added advantage of not only reducing organic load but it also decreases microbial load and infectious aerobic organisms as well as associated odors^16, 17^. These outcomes are achieved through the sequential enzymatic degradation facilitated by rumen derived microorganisms autochthonous to animal manure. These organisms produce enzymes capable of cellulolytic, amylolytic, proteolytic and methanogenic activities. However, in the broad categorization of anaerobic successional processes towards the production of methane biogas, the following sets of biochemical reactions occur, commencing with hydrolysis, which is the degradation of complex macromolecules (carbohydrates, fats and proteins), through the breaking of chemical bonds present to produce monomeric units that are degradable by microorganisms^18, 19^. The microorganisms that have been identified to be responsible for hydrolytic reactions include *Firmicutes* sp., *Fibrobacter* sp. and *Spirochaetes* sp.^20^. The essence of these biochemical reactions is to produce monomers thereby making them biologically available for use as substrate in the second set of biochemical reactions achieved through oxidation and referred to as acidogenesis, which is performed by organisms such as *Clostridium* sp. and *Butyribacterium* sp.^21^ with the intermediate products being volatile fatty acids^22^. This is followed by acetogenesis and the responsible organisms include *Syntrophomonas* sp. and *Syntrophobacter* sp.^23^. This process converts the acids to acetate and H_2_, which are the requisite metabolites (substrates) for the last step, methanogenesis, where organisms such as *Methanoculleus* sp., *Methanobacterium* sp., *Methanobrevibacter* sp. and *Methanosarcina* sp.^24^, produce methane and CO_2_^25^.

It should be noted that each step within these sequential degradation processes is dependent on the presence of the responsible microorganisms and the consequential production of the enzymes at concentrations adequate to achieve the intermediate products for the forward chemical reactions and metabolite production. If the ratios of these enzymes (microorganisms) are reduced within the microbial consortia owing to various exogenous factors, such as, feed nutrient composition (sometimes due to seasonal variations), environmental temperature and pH changes, organic load derived toxicity within the digester, etc., such changes will affect the outcome of the anaerobic digestion process and may reduce the final methane yield^26^. Moreover, other factors such as, species of the livestock and the variable concentrations of nutrients in the livestock feed are known to influence the gut microorganisms and the final consortia composition within the animal manure^27^. Even with the same animal, slight changes in environmental conditions, may routinely vary the microbial consortia found in the animal manure. As such, a clear understanding of evolving microbial diversity provided for in functional metagenomic analysis is important towards elucidating the critical biochemical roles played by the various digesting organisms when the intention of fermentation is to maximize biogas yield from a given substrate^28^.

The overarching objective for commercial fermentation processes is to develop strategies that ensure increased product yield. Some of the critical aspects in biogas production optimisation metrics are process stability, substrate degradation efficiency and gas production kinetics^29^. To improve biodigester performance, co-digestion is a widely accepted optimisation strategy. Typically, co-digestion improves anaerobic digestion optimisation metrics by dilution of inhibitors to levels below minimum inhibitory concentrations (MICs) and/or improves nutritional balances within the substrate, such as the important carbon:nitrogen:phosphorous (C:N:P) ratio necessary to increase microbial proliferation^30, 31^. Pertinent to the fermentation strategy is the prior determination of maximum biogas yield possible from a given amount of mono substrate or mixture (co-digestion) in a near ideal batch fermentation, which can be estimated using the biomethane potential (BMP) laboratory test/assay^32^. Although, BMP assay may not often translate similar outcomes in scaled up semi-continuous (fed-batch) industrial operations where conditions may not be optimal, it remains the standard theoretical framework used in decision making in biogas production^33, 34^. There are other trial and error approaches that have been employed to estimate potential yield, which often rely only on information primarily related to substrates’ nutritional contents and presence of inhibitors for deducing initial experimental ratios^31^. Several of these prediction approaches are considered cheap and provide quick but inaccurate forecasts as compared to BMP. Current theoretical methods for predicting biogas yield do not generally consider stability, degradability, and kinetics, despite the strong influence of microbial activity as well as the associated inhibitory or stimulatory effects to the process. Furthermore, these methods tend not to consider the presence of recalcitrant organics (lignin)^35^.

Understanding that the anaerobic digestion process is facilitated by microbial consortia should therefore direct predictive methods and be integrated into decision making on substrate choices and co-digestion strategies. For example, Matheri et al.^36^ through their experiments showed that chicken manure may assist in stabilizing the abiotics of co-digestion with organic matter from municipal sewage. During co-digestion of swine and cow manures at 28.5 ± 2.5 °C and pH 6.80 ± 0.55, Ogunwande et al.^37^ showed that a ratio of 3:7 respectively had increased biogas yield by 61.5% after 32 days of reaction. These degradation reactions involving microorganisms are executed through a series of interconnected biochemical pathways. Thus, using biochemical pathway predictions, and by reviewing the contributions made by enzymes in the different phases of anaerobic digestion, it is possible to estimate the potential biochemical function exerted by individual groups of microorganisms within consortia^38^, such information is provided by the qualitative and quantitative analysis of functional and active genes in the metagenome of microbial consortia.

The application of bioinformatics tools such as PICRUSt (Phylogenetic Investigation of Communities by Reconstruction of Unobserved States) is very promising as an aid to metagenomic analysis of functional genes from various environmental microbiota^39^. Recently, Wilkinson et al.^40^ used PICRUSt to analyze the biodiversity of the rumen microbiome. In a review by Denman et al.^41^, the use of PICRUSt in the study of rumen microbiome was discussed as a possible strategy for maximizing feeding efficiencies and even lowering methane emissions. Moreover, Lourenco et al.^42^ used PICRUSt for a comparison of the microbiota of the beef cattle both ruminal and fecal. Several authors^43–46^ have specifically focused on its application in biogas processes.

This investigation especially chose the 16S targeted approach for sequencing and obtaining the metagenomic data of bacteria consortia present in the different manure samples to reduce cost with the intention of proffering this approach as routine application for predictive analysis of manure for the purpose of biogas yield potential predictions. It is also noteworthy that the range of bacteria biodiversity is adequately covered using the 16S targeted approach. Although, this approach does not address the archaea population, sufficient information provided from the metagenomic data generated can be used to infer end-product (methane) production, since the metabolites derive in the three preceding stages of anaerobic digestion is what predicts methane production by the slow growing archaea population in AD processes. The present study takes a step further by comparing actual metagenomic data from various animal manures using PICRUSt to predict the possibility of co-digesting substrates for biogas production towards increasing yield. Although, metagenomic analysis provides a comprehensive presentation of microbial biodiversity, it is important to further analyze this information using other bioinformatics statistical tools and a knowledge of the anaerobic process involved in biogas production to make credible inferences. This study intends to present the potential application of PICRUSt in the routine analysis of varied samples of animal manure and to highlight the possibilities of using similar methods for other substrates for increased biogas yield. Such information can be incorporated into BMP models or be included as ancillary correlation to improve predictions. It is hoped that the routine integration of PICRUSt and other genomic functional prediction tools, into yield predictions and consequently process optimization will not only address the short comings of both traditional theoretical BMP prediction models but also provide credible and more accurate information necessary for sound decision making in commercial AD processes. Moreover, this approach may be extended in its application for AD process prediction for sterilized or pre-conditioned manures where the addition of manure is solely for the purpose of providing nutrients rather than as inoculum, where the likelihood of biochemical reactions from one of the manures is considered a possible interference. Overall, this study motivates for the use of bioinformatics prediction tools in routine commercial fermentation processes and its optimization where consortia organisms, functional genes and their enzymes are involved.

## Methodology

### Feedstock characterization and biomethane potentials

The three different types of manure samples were characterised for dry matter (TS) and volatile solids (VS) according to the American Public Health Association (APHA) standard methods for water and wastewater analysis^47^. An ultimate analysis of these manures was also carried out according to the EN15104:2011 standard. Characterisation results were then used to estimate biomethane potentials (BMP) of these substrates according to the methods cited and applied by Rashama et al*.*^48^. These theoretical BMPs were then compared to experimental values reported by other researchers in literature and discussed in the context of manure codigestion strategies.

### Molecular characterisation and sequencing of microbiome from manure samples

Horse, cow, and pig manure samples were collected from different farms as shown in Table 1. All samples were collected in the summer season (February 2020) within an interval of three days and processed for analysis as a batch. The study ensured biological replicates by collecting samples from varied locations. Collected samples were put in sterile plastics and stored in a portable icebox in which they were then transferred to the laboratory for physico-chemical and microbiome analyses. Samples were maintained under refrigeration at 4 °C, not longer than 3 days before analysis.

**Table 1:** Animal manure samples and sources.

Sample	Sources	Sample ID	Location	Description of location and handling of livestock
Cow dung	Boerdery farm produce	C1	− 26.21779, 27.6414784	A farm producing animal feeds as well as rearing cattle fed on grains and concentrates in Randfontein, Gauteng
	Kates farm	C2	− 26.0990952, 27.9103166	A bed and breakfast farm facility in the outskirts of Johannesburg, Gauteng serving beef and chicken from livestock locally reared in a free-range feeding scheme
	Bosheuvel country estates	C3	− 26.0249182, 27.8929324	A farm in the Muldersdrift area of Johannesburg with an on-site vintage hotel, restaurant, event venues and conference rooms serving locally reared Pinzgauer cattle, pigs, chickens, and sheep fed mostly on mixed vegetables and tubbers
Horse manure	Earth centre	H1	− 26.080926, 27.8747353	A non-profit company situated in Ruimsig, Gauteng specialising in Equine Assisted Programmes for children with disabilities.The horses are fed with a wide range of feed concentrates and probiotics
	Harveston stables	H2	− 26.0990952, 27.9103167	A yard in Honeydew, Gauteng offering horse riding lessons, stabling, kids pony parties, picnics and pony rides. The horses are fed mainly on feed concentrates
	Barent horse stables	H3	− 26.21779, 27.6414783	A plot rearing horses for family leisure in Randfontein, Gauteng. The horses are fed mostly on lucerne
Pig manure	Bosheuvel country estates	P1	− 26.0249182, 27.8929324	A farm in the Muldersdrift area of Johannesburg with an on-site vintage hotel, restaurant, event venues and conference rooms serving locally reared Pinzgauer cattle, pigs, chickens, and sheep fed mostly on mixed vegetables and tubbers
	Bosheuvel country estates	P2	− 26.0249182, 27.8929324	
	Country portion farm	P3	− 26.224517, 27.6325603	A farm in Randfontein producing vegetables, poultry, and pork products to supply local restaurants and consumers. The poultry and pigs are fed on corn and sorghum and concentrates

Genomic DNA was extracted from the manure samples using the DNeasy PowerSoil kit (QIAGEN, Germany), following the manufacturer’s instructions. These samples were analysed in triplicate. Amplification of genomic DNA extracts employed polymerase chain reaction (PCR) using the universal bacterial primers, 27 F (5′-AGAGTTTGATCMTGGC-3′) and 518 R (5′-GTATTACCGCGGCTGCTGG-3′) targeting the conserved bacterial 16S rRNA gene as described by Selvarajan et al.^49^.

PCR products purification was achieved using DNA Clean & Concentrator Kit (ZYMO RESEARCH, Irvin, USA). Prior to the library preparation and sequencing process, the triplicate samples from individual locations were pooled together before Illumina sequencing adapters and dual-index barcodes were added to the amplicon targets using full complement of Nextera XT indices (Illumina, Inc. San Diego, CA, USA) followed by an 8-cycle PCR run (95 °C for 3 min, 95 °C for 30 s, 55 °C for 30 s, and 72 °C for 30 s, with a final extension at 72 °C for 5 min, then cooling at 4 °C). Further cleaning of PCR products utilised AMPure XP beads. A bioanalyzer DNA 1000 chip (Agilent, Santa Clara, CA, USA) was then used to validate the fragments size (~ 630 bp) before quantifying them in a fluorometric quantification method (Qubit, USA) integrating dsDNA binding dyes for visualization. Dilutions were achieved on the quantified DNA using 10 mM Tris Buffer (pH 8.5). 5 μl of diluted DNA was aliquoted from each library and mixed for pooling libraries. The pooled final DNA library (4 nM) was denatured and sequenced on an Illumina Miseq System. Sequencing employed paired 300-bp reads to generate high-quality, full-length reads of the V3 and V4 regions. Raw fastq sequence files were then obtained after trimming the adapters and primer sequences. Sequences derived were subjected to bioinformatics analyses.

### Bioinformatics analyses

The fastq sequence files from the Miseq were inspected for quality using the FastQC software (v 0.11.7, Babraham Bioinformatics, United Kingdom). Using PANDAseq.37 on the QIIME2 platform, the forward and reverse reads were merged and then clustered into operational taxonomic units (OTUs) at a sequence similarity of 97% for species level identification using the ‘pick_open.reference_otus.py’ script in QIIME, while aligning against the SILVA rRNA (release 132) database38 by using usearch61 and PyNAST aligner. The OTU table generated was exported to the R-studio for further statistical analyses. R packages: vegan, ape, labdsv and ggplot were installed and used for statistical analyses and plotting^50^.

### Taxonomic identification and prediction of functional gene content

To assess the metabolic potential of microbial communities found in the three different animal manures, 16S rRNA sequence reads were clustered into operational taxonomic units (OTUs) using the closed-reference method in QIIME2 software. The generated OTU table was imported into the PICRUSt and the Kyoto Encyclopaedia of Genes and Genomes (KEGG) database was used to predict the functional gene content of the various microbial communities represented in the Greengenes database of 16S rRNA gene sequences^51^.

The output from KEGG database containing the predicted function was further analysed using Linear discriminant analysis (LDA) combined with linear discriminant analysis effect size (LEfSe). The purpose of this was to identify the most differentially abundant predictions among three different animal manure samples. LEfSe was used online in the Galaxy workflow framework. The predicted functions output from KEGG was also exported to the R-studio for further statistical analysis. The R packages vegan, ape, heatmap.plus, ggplot2 and readxl were installed and used for statistical analysis as well as plotting. STAMP version 2.1.3 was also used as a graphical tool.

## Results

### Manure characterisation and biomethane potential calculations

The characterisation and BMP results for the three (3) types of manures considered in this study are reported in Table 2 with calculations described in Supplementary Data Table S1. For each manure, three theoretical BMP calculation approaches were used, and two results based on previous laboratory assays were also reported for each substrate.

**Table 2:** Substrate physicochemical properties and biomethane potentials.

Parameter (units)	Cow dung	Pig manure	Horse manure
TS (% of wet mass)	14.5 (1.7)	20.4 (2.4)	21.8 (0.8)
VS (% of TS)	88.7 (2.1)	82.8 (2.8)	86.8 (2.4)
C (% of TS)	25.7 (8.3)	27.2 (6)	30.6 (5.7)
H (% of TS)	3.8 (0.8)	4.1 (0.4)	4.2 (0.4)
N (% of TS)	4.9 (1.6)	2.1 (1)	1.5 (0.4)
S (% of TS)	0.2 (0.1)	0.2 (0.0)	0.2 (0.1)
O (% of TS)	54.0 (7.8)	49.2 (8.4)	50.3 (6.7)
Empirical formula	C_307_H_547_O_404_N_50_S	C_330_H_590_N_22_O_447_S	C_396_H_649_N_17_O_488_S
ThOD (gO_2_/gVS)	0.41	0.63	0.72
HHV (MJ/t)	4626.8	6287.4	7393.0
BMP_F_ (ml CH_4_/gVS)	144.8	221.6	251.4
BMP_B_ (ml CH_4_/gVS)	144.0	220.6	250.4
BMP_D_ (ml CH_4_/gVS)	122.5	166.4	195.7
BMP_L1_ (ml CH_4_/gVS)	204 (12)	155 (2)	323 (13)
BMP_L2_ (NL CH_4_/kgVS)	10.44	14.5	17.09

L1 Reference^52^.

L2 References^53^.

Physico-chemical results and BMP_L1_ are reported as means ± (standard deviations), n = 3.

*ThOD* theoretical oxygen demand, *HHV* higher heating value, *VS* volatile solids; *TS* total solids, *F* Forgacs approach, *B* Buswell approach, *D* Dulong approach.

### Taxonomic identification

**Figure 1:**
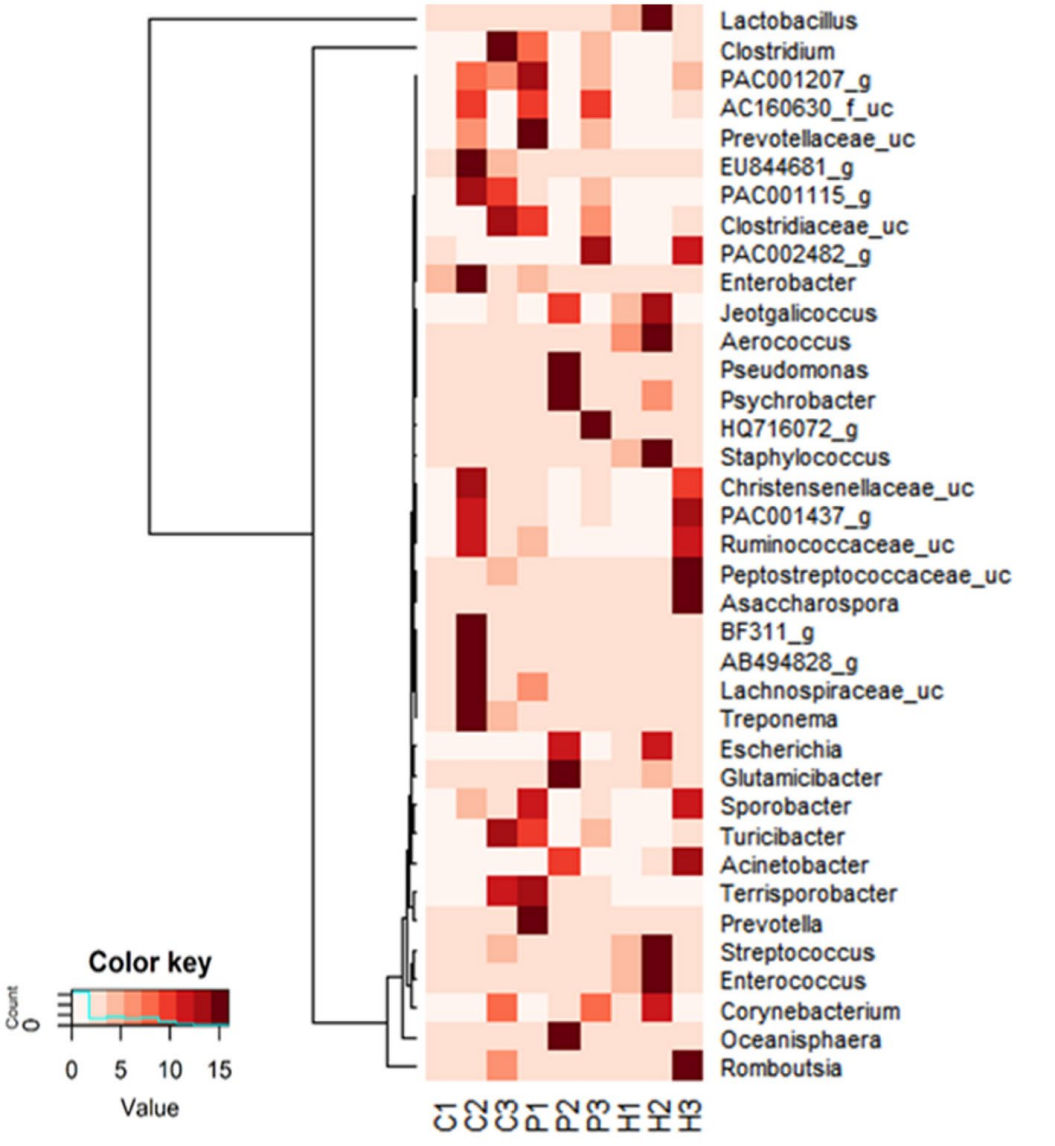
Heat map showing the relative abundance of microorganisms in different animal manures.

Genus level diversity analyses in manure samples were performed with quality checks implemented on the Silva rRNA gene database at the cut-off level of 97% similarities. The metagenomic qualitative and quantitative analyses of samples have been included in the supplementary data Table S2. The prevalence and relative abundance of the genera in all 9 samples of manure is shown in Fig. 1 as represented with a heat map of bacterial genus distribution and prevalence. Bacteria population distribution showed similarities for some of the different samples, however at varied abundance, it is also showed the presence of genera that were unique to only one manure type but not found in other samples, the specific distribution and predicted metabolic contributions of each of the relevant genera to biogas production are described in Table 3.

**Table 3:** Identified genera in animal manure samples and their predicted metabolic contributions to biogas production.

Genus	Relative abundance in animal manure	Predicted metabolic contribution	References
**Bacteria genera in consortia likely playing roles in pathways related to growth and the synthesis and degradation of growth metabolites in manure**			
*Escherichia*	Horse < cow < pig	Facultatively anaerobic with wide spectrum capability for several organic carbon sources necessary for heterotrophic growth, also known to be involved in acidogenesis. Mostly harmless but some species and strains are pathogenic	^57, 58^
*Glutamicibacter*	Horse < cow < pig	Members of the genus are involved in lignocellulosic material saccharification, amino acid metabolism	^59, 60^
*Psychrobacter*	Pig < Horse < cow	*Psychrobacter* spp. are capable of producing cold-active enzymes with involvement in the physiological strategies that off-set low temperature effects on cellular ATP and ADP generation, a key requirement in metabolic and energy conservation reactions The KEGG pathway shows involvement in riboflavin and tryptophan metabolism (see *P. cryohalolentis* and *P. articus*)	^61^
*Aerococcus*	Horse < cow < pig	Members of the genus produce acids from a variety of carbohydrates and are directly involved in acidogenesis The KEGG pathway shows involvement of A. urinae in anaerobic energy and selenocompound metabolism. Some species are considered pathogenic	^62–64^
*Oceanisphaera*	Pig	The KEGG pathway shows involvement of *Oceanisphaera profunda* in selenocompounds, arachidonic and 2-oxocarboxylic acid metabolism	^65^
*Turicibacter*	Horse = pig = cow	Members of the genus have been identified as being involved in fat metabolism e.g. *T. sanguinis*, might be important for host lipid and steroid metabolism The KEGG pathway shows the involvement of *Turicibacter* sp. H121 in Tryptophan metabolism and *T. sanguinis* involvement in carbohydrate, amino acid, lipid and nucleic acid metabolism	^66–68^
*Romboutsia*	Pig < cow = horse	Members of the genus have a broad range of capabilities in carbohydrate utilisation but not necessarily cellulose and xylose, fermentation of single amino acids, anaerobic respiration and metabolic end products. Although, there are variations in these abilities with different strains in the manner in which they utilise carbohydrates to synthesize vitamins and nitrogen as well as nitrogen assimilation capabilities	^69, 70^
**Bacteria genera in consortia likely playing roles in pathways related to complex lignocellulose degradation and represented within other hydrocarbon pathways**			
*Glutamicibacter*	Horse < cow < pig	Members of the genus are involved in lignocellulosic material saccharification, amino acid metabolism	^59, 71^
*Jaetgalicoccus*	Horse < pig < cow	Members of the genus are capable of producing terminal alkenes inferring its production of functional enzymes in complex hydrocarbon degradation. It produces enzymes involved the one-step fatty acid decarboxylation reaction employing OleTJE cytochrome P450. KEGG pathway describes thiamine metabolism in *Jeotgalicoccus* sp. ATCC 8456	^72, 73^
**Bacteria genera in consortia likely playing roles in pathways with direct relation to acidogenesis and acetogenesis**			
*Escherichia*	Horse < cow < pig	Facultatively anaerobic with wide spectrum capability for several organic carbon sources necessary for heterotrophic growth, also known to be involved in acidogenesis. Mostly harmless but some species and strains are pathogenic	^57, 74^
*Jaetgalicoccus*	Horse < pig < cow	Members of the genus are capable of producing terminal alkenes inferring its production of functional enzymes in complex hydrocarbon degradation. It produces enzymes involved the one-step fatty acid decarboxylation reaction employing OleTJE cytochrome P450. KEGG pathway describes thiamine metabolism in *Jeotgalicoccus* sp. ATCC 8456	^72, 73^
*Aerococcus*	Horse < cow < pig	Members of the genus produce acids from a variety of carbohydrates and are directly involved in acidogenesis The KEGG pathway shows involvement of *A. urinae* in anaerobic energy and selenocompound metabolism. Some species are considered pathogenic	^62–64^
*Enterococcus*	Horse < pig < cow	Members of the genus employ fermentative metabolism for the conversion of a variety of carbohydrates to lactic acid. They are strict anaerobes as they lack apparatus for implementing Kreb’s cycle reactions. However, they utilise each of the three possible routes of intermediary carbohydrate metabolism – the Embden-Meyerhof-Parnas (glycolysis), Entner-Doudoroff, and pentose phosphate (phosphogluconate) pathways	^75, 76^
*Staphylococcus*	Horse < pig < cow	Some members of the genus *Staphylococcus* are facultatively aerobe and in aerobic conditions can synthesize enzymes such as lactate dehydrogenases and alcohol dehydrogenases with an accumulation of lactic acid and acetic acid. In hypoxic conditions they associate and form biofilms for protection	^77, 78^
*Lactobacillus*	Horse = pig < cow	Lactobacilli ferment hexose sugars to produce lactic acid using the phosphoketolase pathway to produce lactate, CO_2_ and acetate or ethanol as major end products. They are also capable of acidogenesis biosyntheses of amino acids, purine/pyrimidines, and cofactors	^79, 80^
*Corynebacterium*	Horse < cow = pig	Corynebacteria demonstrate fermentative metabolism of various carbohydrates to lactic acid under certain conditions. They are fastidious slow-growing organisms that are also able to produce glutamic acid, lysine and threonine. The KEGG pathway describes nitrogen metabolism in *C. glutamicum* R	^81^
*Prevotella*	Pig = cow	Members of this genus utilise glucose in anaerobic growth using the Embden-Meyerhof-Parnas pathway and the usual enzymes involved except that phosphofructokinase was pyrophosphate-dependent. The cells use available glucose to produce acetate, formate and succinate	^82, 83^
*Terrisporobacter*	Pig = cow	Members of the genus are able to ferment glucose to produce acetates	^84, 85^
*Streptococcus*	Horse < cow = pig	Species of *Streptococcus* utilise carbohydrate metabolism to generate energy for growth generating acids in the process. They are mostly pathogenic	^86, 87^
*Clostridium*	Cow < Pig < horse	Most members of these species are pathogenic to animals. They are capable of converting various carbohydrates to succinate and acetate	^70, 88^
*Sporobacter*	Horse = pig	Members of the genus are capable of using organic compounds in metabolism yielding acetates	^89, 90^
**Bacteria genera in consortia that are likely pathogenic**			
*Asaccharospora*	Horse	One identified species *Asaccharospora irregularis* resembling in characteristics *Clostridium irregularis* described as pathogenic	^70^
*Aerococcus*	Horse < cow < pig	Members of the genus produce acids from a variety of carbohydrates and are directly involved in acidogenesis The KEGG pathway shows involvement of A. urinae in anaerobic energy and selenocompound metabolism. Some species are considered pathogenic	^62–64^
*Acinetobacter*	Horse = pig < cow	Most members of this genus are pathogenic and possess virulence factors but enzymes produced are also involved in amino acid, carbohydrate and lipid transport and metabolism	^91^
*Pseudomonas*	Horse = pig < cow	*Pseudomonas* spp. perform anaerobic energy metabolism, carbon-sources versatility observed in the free-living bacteria allowing it to selectively assimilate a preferred carbon-source from mixtures in a process known as carbon catabolite repression using regulatory mechanisms. Some species have multiple virulence factors	^92, 93^
*Streptococcus*	Horse < cow = pig	Species of *Streptococcus* utilise carbohydrate metabolism to generate energy for growth generating acids in the process. They are mostly pathogenic	^86, 87^
*Clostridium*	Cow < Pig < horse	Most members of these species are pathogenic to animals. They are capable of converting various carbohydrates to succinate and acetate	^70, 88^
*Treponema*	Cow	Members of the genus are pathogenic	^94, 95^

### Prediction of functional gene content

**Figure 2:**
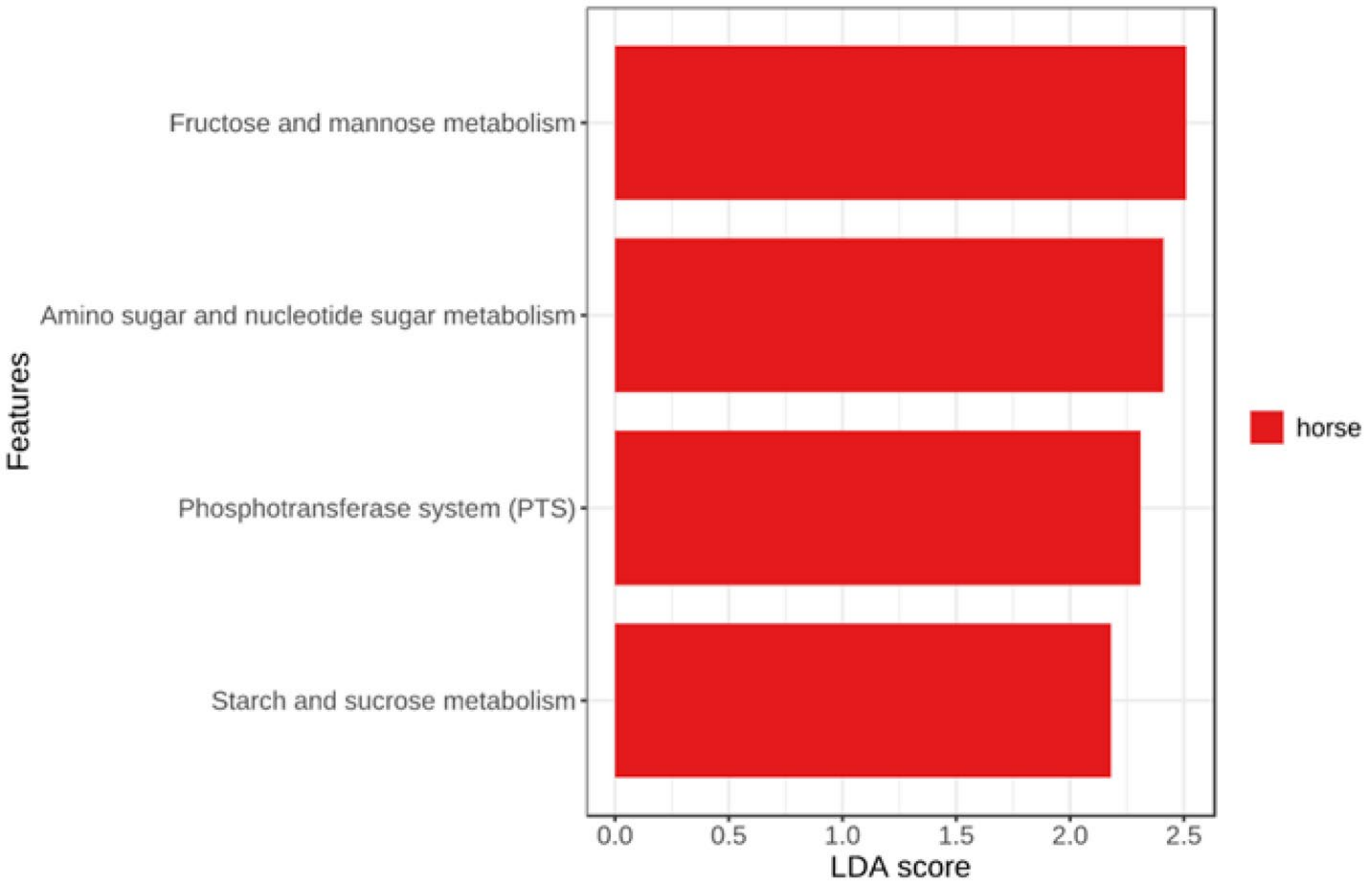
Linear discriminant analysis (LDA) combined with effect size measurements (LEfSe). Histogram of the LDA scores computed for differentially abundant predicted functions among the three animal manure samples. A p-value of < 0.05 and 2.0 or higher LDA score were considered significant in Kruskal–Wallis.

The BIOM format OTU table from QIIME2 was processed on PICRUSt using the KEGG database. A total of 135 predicted KOs (KEGG Orthologies) were grouped into level 2 of categorization. Comparison of metabolism showed four major categories of metabolic activities see Fig. 2. Other classes of metabolism such as carbohydrate, energy, lipid and xenobiotic biodegradation and metabolisms were well represented in each manure sample, with horse manure showing high representation (Supplementary Table S3).

**Figure 3:**
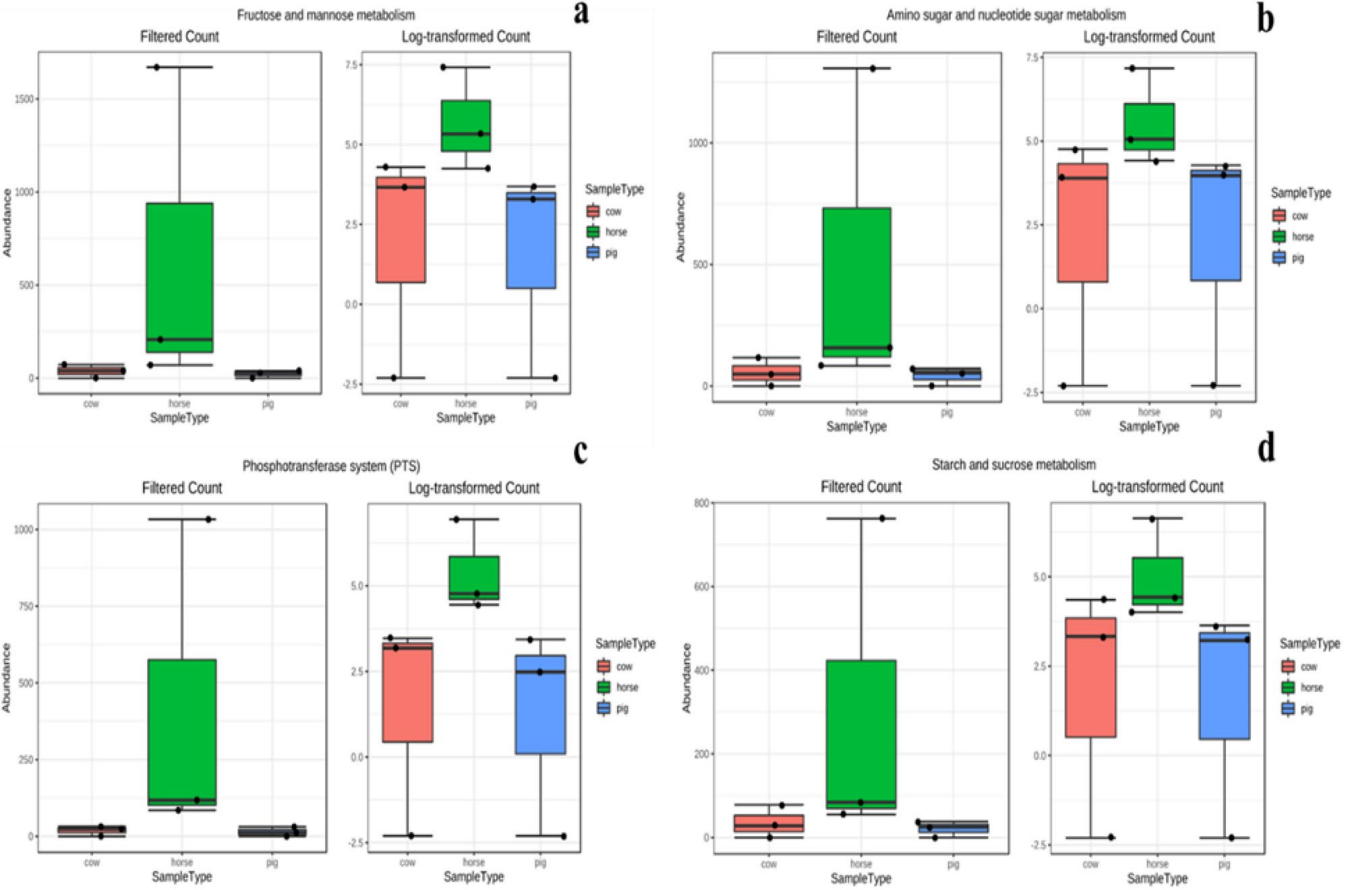
Significant features highlighted in LDA (linear discriminant analysis) LefSe = LDA effect size. (**a**) Fructose and mannose metabolism (KO 00051), (**b**) amino acid and nucleotide sugar metabolism (KO 00520), (**c**) phosphotransferase PST pathway (map02060) and (**d**) starch and sucrose metabolism (KO 00500).

Results obtained from linear discriminant analysis (LDA) combined with the effect size measurements (LEfSe), where the comparison and identification of the predicted functions that were significantly different among the three different animal manures, showed four significant features in horse manure samples. These significant features include KEGG pathways for fructose and mannose metabolism, amino acid and nucleotide sugar metabolism, phosphotransferase PST as well as starch and sucrose metabolism (Fig. 2). The abundance of each significant feature in horse manure, compared to cow and pig manure, was represented using boxplot (Figs. 3a–d).

**Figure 4:**
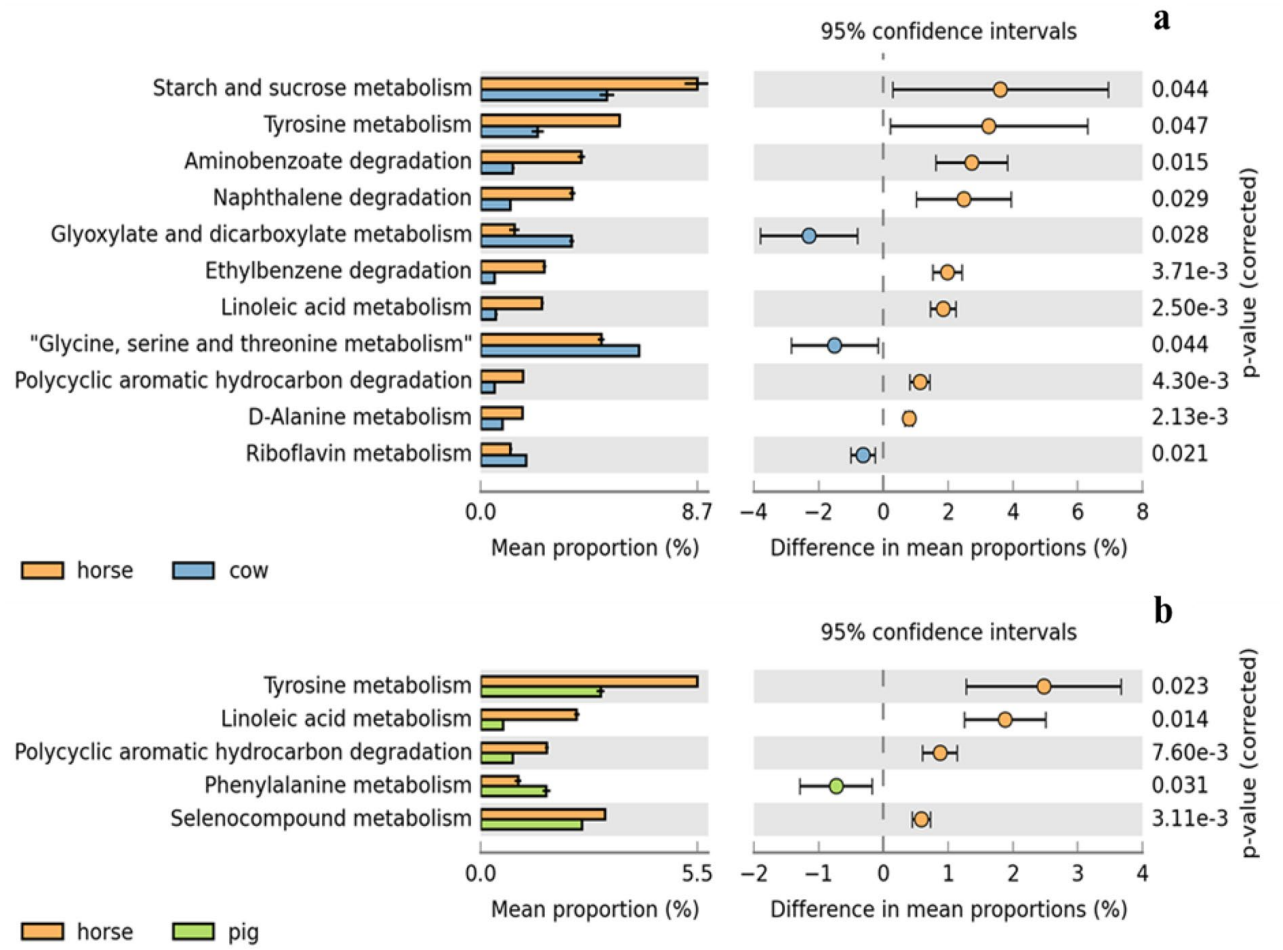
Extended error bar plot for two-group analysis module comparison of PICRUSt predicted KEGG function data based on horse-cow (**a**) and horse-pig (**b**) using Welch’s t-test for two groups. An extended error bar plot was used for the comparison between 2 manure samples and only predicted functions with p < 0.05 are shown. Bar plots on the left side display the mean proportion of each KEGG pathway while the dot plots on the right show the differences in mean proportions between 2 manure samples using p-values.

The PICRUSt approach was used to evaluate the functional potential of microbial communities with a particular focus on biogas production. The obtained predicted functions were therefore selected based on their relevance to anaerobic digestion. Out of the 135 predictions, 36 were related to the second or third stage of anaerobic digestion, acidogenesis or acetogenesis. The differences in the number of predictions among the biological replicates of the manure samples obtained from various locations as previously described in Table 1 and represented by the extended bar plot (Figs. 4a,b). The latter compared horse-cow manure, KEGG pathway predictions showed a total of 11 significant predictions (Fig. 4a). While comparison based on horse-pig showed 5 significant predictions (Fig. 4b). There were no significant predictions in the comparison of cow-pig manure. The relative proportions of all the 36 KEGG pathway predictions is shown in S2.

### Correlation analysis of the genus distribution of bacteria in different animal manures

Table 3 is derived from the combined analysis of Fig. 1 in relation to the PICRUSt analysis of predicted functions shown in Figs. 2 and 4 and Supplementary Data Tables S3. Bacteria genus identified were searched from previous literature that identified related functions relevant to metabolic functions of biogas production in focus for this study. Applying KEGG pathway predictions (Fig. 4a), showed that a total of 11 significant predictions were observed compared with horse-cow manure. While comparison based on horse-pig showed 5 significant predictions (Fig. 4b). There was no significant prediction in the comparison of cow-pig manure. Therefore, the correlation analysis presented in Table 3 may be loosely classified as follows:

1. pathways related to growth and the synthesis and degradation of growth metabolites. Such as that provided by linoleic acid and amino acids including, tyrosine, glycine, serine, threonine, d-Alanine metabolism and phenylalanine, as well as in the metabolism of riboflavin and selenocompounds^54, 55^;
2. pathways related to complex lignocellulose degradation as represented within other hydrocarbon pathways of Aminobenzoate, Naphthalene, Ethyl benzene and PAH degradations^54, 55^;
3. pathways with direct relationships to acidogenesis and acetogenesis such as those represented with starch and sucrose metabolism^54, 55^. This is related to the transformation of by-products to short-chain (C1–C5) organic acids, alcohols, hydrogen, and carbon dioxide^28^. Glyoxylate and dicarboxylate metabolism pathways are also present, which are major reactions involved for acetogenesis^56^.

These functions were predominantly observed in manure samples obtained from the horse, with remarkable functions of riboflavin metabolism observed in cow to be high, while phenylalanine and selenocompound metabolism was prevalent in pig manure samples. Although, not related directly to biogas production a fourth classification incorporated all pathogenic bacteria genus that did not necessarily contribute to metabolic function but were significantly present in the different manure samples.

## Discussion

The mono-digestion biomethane potential of the manures increased in the order of cow, pig and horse based on three predictive models that use the ultimate analyses of the substrates studied. This implies that co-digesting with the highest BMP producing manure, the horse manure, can result in the highest BMP for the resulting mixture using the particular samples from this study. However, this hypothesis may fail if the relevant microbial diversity in the digesters is not balanced to drive the anaerobic digestion processes that result in generation of methane. The current microbial based inference is in agreement with the high BMP of horse manure among the three manure candidates when used as co-substrates. The mono-digestion data also corroborate with our findings where horse manure gave the highest BMP among the three manures.

While investigating the mono-substrate BMPs, Castro-Molano et al.^52^ studied the co-digestion of these manures in a laboratory BMP assay and established that all mixtures (co-digestions) using different ratios had synergies above the value of 1 with the highest synergy being reported for cow-horse mixture. All co-digestions involving cow-pig-horse manures gave higher BMP values than any single manure, thus indicating the synergistic benefits of co-digestions and selection of the best co-substrate. In an independent co-digestion study, Alfa et al.^96^ showed that a mixture of cow and horse manure had a higher BMP than any of the two reacted separately. A mixture of pig and horse manure also gave a higher BMP than for the two substrates separately^97^. The researchers in this investigation attributed the high BMP registered on co-digestion to the synergistic effects of both a richer microbial diversity and balanced nutritional ratios.

In this study, the microbial community structure was analysed using Illumina amplicon sequencing of the 16S rRNA gene. This limited the scope of investigation to bacteria and their roles in the stepwise processes of anaerobic digestion, thus focusing the study on biochemical pathways that are predominantly the domain of bacteria, hydrolysis, acidogenesis and acetogenesis. Acidogenesis and acetogenesis provide the substrates (metabolites) utilised by the predominantly archaea methanogen communities that are pivotal to the last step of bio methanation. It is known that the archaea methanogens transform the products of acetogenesis (acetate, carbon dioxide and hydrogen) into methane^98, 99^, via interspecies hydrogen and acetate transfer. This is considered the determining factor controlling the completion of anaerobic digestion^100, 101^. Ensuring the adequate availability of metabolites, and an enabling anoxic environment that are requisites for the growth of methanogens and ensure high biogas yield within the biodigester. This implies that the three stages (hydrolysis, acidogenesis and acetogenesis) essentially direct yield for the slow growing methanogens. As such, this study motivates that the 16S rRNA functional characterisation is more than adequate from a cost-saving perspective for correlation analysis and trouble-shooting protocols to improve biogas yields during anaerobic digestion.

The rather extensive feature of the KEGG database indicates a requirement for selective analysis using the combination of LDA and LEfSe to reduce the scope of analysis and ease the identification of the most differentially abundant predictions (Figs. 2, 3). Moreover, the high variations observed in Figs. 3 and 4 are expected in analysis dealing with biological replicates and microorganisms with their varied functions, features and adaptations^102–104^ which are the consequences of the different environments from which they originated as described in Table 1.

However, the significant features identified from the KEGG pathways were fructose and mannose metabolism, amino acid and nucleotide sugar metabolism, phosphotransferase PST as well as starch and sucrose metabolism (Fig. 2) and all of which are linked to hydrolysis and initial break down of complex compounds and substrates. All these pathways utilise hydrolytic processes but pertinently provide the ATP and biomolecules required in anaerobic digestion and biogas production. Remarkably, the abundance of these significant pathways was observed to be the most in prevalence in horse manures in comparison to cow and pig manure samples (Fig. 3a–d).

A study on the comparative daily feed intake of horses versus cows by Menard et al.^105^ highlighted that on average, horses consume 144 g DM kg W^−0.75^ day^−1^ while cows consume 88 g DM kg W^−0.75^ day^−1^. This implies that horses consume 63% more forage than cows. The same study also discovered that the daily intake of digestible dry matter (nutrient extraction) in all seasons was considerably higher in horses (78 g DM kg W^−0.75^ day^−1^) than in cattle (51 g DM kg W ^−0.75^ day^−1^) indicating a higher functional response in horses than in cattle. It may thus be inferred that the horse GIT has a higher enzymatic capacity to degrade feed faster than the cow and pig GITs. This could explain the prediction of higher fructose, mannose, amino, nucleotide sugar, phosphotransferase PST as well as starch and sucrose metabolism in horses than in cows and pigs. Therefore, the use of horse manure showing this feature can be employed to achieve the initial degradation of complex structured sugars within a biodigester to monomeric biomolecules for other microorganisms to process further.

Table 3 provides a review of the predominantly identified genera of microorganisms found in each manure (derived from Fig. 1), but further provides a brief description of the major functions reported in various literature and the KEGG pathways for previously identified species^54, 55^. This table buttresses the relationship between the PICRUSt functional predictions, and the identified microorganisms present within manure samples. In Table 3 organisms are placed in this identified classification obtained from the derived PICRUSt functions. Table 3 allows for a panoramic overview making it possible to infer likely functions and contributions by enzymes from the genera in the consortia present in respective animal manures. The emphasis, however, was on the likely roles within the biodigester and anaerobic digestion to produce biogas, therefore a necessary sifting of available literature is necessary when conducting such an analysis.

The analysis made it possible to identify which functions are present or lacking within each manure and provides the possibility of increasing the quantities of a particular type of manure in a co-digestion strategy to compensate for any inadequate microbial genus representations needed for the anaerobic digestion process. For example, it was observed in Table 3 that there was a predominance of most of the relevant microorganisms for all related functions of AD in horse manure, the implication to co-digestion ratios is that it needs to consider increasing the quantities of manure obtained from the horse against the quantities of the other two (cow and pig).

Similarly, should the biodigester be operated in winter conditions, it might benefit from an increase in pig manure to increase the bacterial load of *Psychrobacter* with its propensity to produce cold active enzymes that off-set low temperature effects on cellular ATP and ADP generation^61^. Low temperatures have been identified as a challenge in operating biodigesters^106, 107^. Although, this might be considered small changes, but BMP would not provide such nuanced information, and this makes a significant difference to the process kinetics in this particular scenario. It should also be noted that the microbial profile composition for this study in this context only lends to these two particular changes, but different profiles will provide, after analysis their unique set of changes necessary to make for better process kinetics.

Conversely, the study identified a significant presence of several pathogenic genera including, *Asaccharospora, Aerococcus, Acinetobacter, Pseudomonas, Streptococcus, Clostridium, Treponema*. This is not surprising but also brings to cognizance the need for an anaerobic digestion treatment of manure before use so as to reduce the majority of the likely zoonotic pathogens present in the different samples of animal manure, preventing its possible transmission to humans^108^. Thus, reaffirming anaerobic digestion as a useful pre-treatment of manure before use as soil amendment or as fertilisers.

## Conclusion

While this study focused on three different types of animal manures, it highlighted the complex variations of bacterial diversity and the likely functional contributions possible from the bacterial communities. However, this diversity is subject to constant changes due to several factors including seasonal variations, temperature, pH and nutrition. Viable commercial process will require routine evaluation and analysis to determine optimal strategies of co-digestion to maximise the yield of biogas. Moreover, such information can be useful in trouble shooting exercises to determine system failures as it relates to abundance or deficiencies in bacteria population and their effects in an operating biodigester for biogas production.

## Supplementary Information

Supplementary Information.

## Data Availability

The authors confirm that the data supporting the findings of this study are available within the article. The raw genomic sequence data used was generated at UNISA in CAES and has been uploaded to an NCBI repository and with other pipelines used during bioinformatics analysis, also all data are available from the corresponding author on request.
