# Supplementary material for: Applying PICRUSt and 16S rRNA functional characterisation to predicting co-digestion strategies of various animal manures for biogas production
Source: Sci Rep. 2021 Oct 7;11:19913. doi: 10.1038/s41598-021-99389-4 (PMC8497515; doi:10.1038/s41598-021-99389-4)
Supplement: Supplementary file 1 — Supplementary Information. [file 41598_2021_99389_MOESM1_ESM.docx]

**Applying PICRUSt and 16S rRNA functional characterisation to predicting co-digestion strategies of various animal manures for biogas production**

Grace N. Ijoma^1*^, Rosina Nkuna^1^, Asheal Mutungwazi^1^, Charles Rashama^1^, Tonderayi S. Matambo^1^

1. Institute for the Development of Energy for African Sustainability, University of South Africa, Christiaan De Wet/Pioneer Dr. P.O. Box X6, Florida, 1710, South Africa

## Supplementary Information

**Corresponding Author:**

G.N., Ijoma

Institute for the Development of Energy for African Sustainability,

University of South Africa,

28 Pioneer Ave, Florida Park,

Roodepoort, 1709

South Africa

Email: [nkechiijoma@gmail.com](mailto:nkechiijoma@gmail.com)

Telephone: +27 11-6709049

**Table S1: Biomethane Potential (BMP) calculations for different manure samples**

**AS % of VS AS % of TS**

| **Manure Type** | **Location** | **Sample ID** | **VS %** | **%C** | **%H** | **%N** | **%S** | **%O** |  | **%C** | **%H** | **%N** | **%S** | **%O** |
| --- | --- | --- | --- | --- | --- | --- | --- | --- | --- | --- | --- | --- | --- | --- |
| Cow dung | Boerdery Farm | C1 | 86,3 | 28,44222 | 4,037479 | 5,715109 | 0,223371 | 61,58182 |  | 24,546 | 3,484 | 4,932 | 0,193 | 53,145 |
| Cow dung | Kates Farm | C2 | 89,5 | 38,54454 | 5,316199 | 3,7408 | 0,217371 | 52,18109 |  | 34,497 | 4,758 | 3,348 | 0,195 | 46,702 |
| Cow dung | Bosheuvel Country Estates | C3 | 90,2 | 19,96905 | 3,561827 | 7,171597 | 0,313245 | 68,98428 |  | 18,012 | 3,213 | 6,469 | 0,283 | 62,224 |
| Chicken manure | Kates Farm | F1 |  | 38,75867 | 5,345733 | 1,750471 | 0,218578 | 53,92655 |  | 0,000 | 0,000 | 0,000 | 0,000 | 0,000 |
| Chicken manure | Country Portion Farm | F2 |  | 23,70409 | 3,105783 | 9,800594 | 0,2511 | 63,13844 |  | 0,000 | 0,000 | 0,000 | 0,000 | 0,000 |
| Chicken manure | Bosheuvel Country Estates | F3 |  | 33,02222 | 4,817961 | 1,692034 | 0,205244 | 57,26254 |  | 0,000 | 0,000 | 0,000 | 0,000 | 0,000 |
| Pig manure | Bosheuvel Country Estates | P1 | 85,3 | 26,65819 | 4,262011 | 2,671156 | 0,292248 | 66,1164 |  | 22,739 | 3,635 | 2,278 | 0,249 | 56,397 |
| Pig manure | Bosheuvel Country Estates | P2 | 83,4 | 29,86316 | 4,958973 | 3,632597 | 0,233624 | 61,31164 |  | 24,906 | 4,136 | 3,030 | 0,195 | 51,134 |
| Pig manure | Country Portion Farm | P3 | 79,7 | 42,75698 | 5,518987 | 1,342751 | 0,271066 | 50,11022 |  | 34,077 | 4,399 | 1,070 | 0,216 | 39,938 |
| Horse manure | EARTH Centre | H1 | 89,3 | 37,96905 | 5,561827 | 2,171597 | 0,313245 | 53,98428 |  | 33,906 | 4,967 | 1,939 | 0,280 | 48,208 |
| Horse manure | Harveston Stables | H2 | 84,5 | 40,21379 | 5,251833 | 1,263075 | 0,175964 | 53,09534 |  | 33,981 | 4,438 | 1,067 | 0,149 | 44,866 |
| Horse manure | Barent Horse Stables | H3 | 86,7 | 27,67282 | 3,626773 | 1,848779 | 0,219147 | 66,63249 |  | 23,992 | 3,144 | 1,603 | 0,190 | 57,770 |

**Calculations**

**Moles Mole Ratios**

Cow Pig Horse Cow Pig Horse

Cow dung Pig manure Horse manure manure manure manure manure manure manure

| C | Mean | 25,7 | 27,2 | 30,6 |  | 2,140 | 2,270 | 2,552 |  | 307 | 330 | 396 |
| --- | --- | --- | --- | --- | --- | --- | --- | --- | --- | --- | --- | --- |
|  | SD | 8,3 | 6,0 | 5,7 |  |  |  |  |  |  |  |  |
| H | Mean | 3,8 | 4,1 | 4,2 |  | 3,818 | 4,057 | 4,183 |  | 547 | 590 | 649 |
|  | SD | 0,8 | 0,4 | 0,4 |  |  |  |  |  |  |  |  |
| N | Mean | 4,9 | 2,1 | 1,5 |  | 0,351 | 0,152 | 0,110 |  | 50 | 22 | 17 |
|  | SD | 1,6 | 1,0 | 0,4 |  |  |  |  |  |  |  |  |
| S | Mean | 0,2 | 0,2 | 0,2 |  | 0,007 | 0,007 | 0,006 |  | 1 | 1 | 1 |
|  | SD | 0,1 | 0,0 | 0,1 |  |  |  |  |  |  |  |  |
| O | Mean | 54,0 | 49,2 | 50,3 |  | 3,376 | 3,072 | 3,143 |  | 484 | 447 | 488 |
|  | SD | 7,8 | 8,4 | 6,7 |  |  |  |  |  |  |  |  |
|  |  |  |  |  |  |  |  |  |  |  |  |  |
| Empirical Formula | | C_307_H_547_O_484_N_50_S | C_330_H_590_N_22_O_447_S | C_396_H_649_N_17_O_488_S |  | 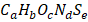 |  |  |  |  |  |  |
| ThOD | | 0,41369672 | 0,633257463 | 0,718239456 |  |  |  |  |  |  |  |  |
| HHV (Dulong) | | 4626,792375 | 6287,353832 | 7393,018121 |  |  |  |  |  |  |  |  |
| Forgacs BMP | | 144,8 | 221,6 | 251,4 |  |  |  |  |  |  |  |  |
| Buswell BMP | | 144,0 | 220,6 | 250,4 |  |  |  |  |  |  |  |  |
| Dulong BMP | | 122,5 | 166,4 | 195,7 |  |  |  |  |  |  |  |  |

**Table S2: Relative abundance of 16S rRNA for different manure samples**

| **Taxon Name** | **Cow1** | **Cow2** | **Cow3** | **Pig1** | **Pig2** | **Pig3** | **Horse1** | **Horse2** | **Horse3** |
| --- | --- | --- | --- | --- | --- | --- | --- | --- | --- |
| Lactobacillus | 63 | 22 | 32 | 62 | 5327 | 2281 | 15606 | 63726 | 0 |
| Clostridium | 5 | 49 | 23951 | 13218 | 21 | 4789 | 38 | 23 | 647 |
| Romboutsia | 0 | 0 | 2309 | 195 | 1 | 217 | 34 | 177 | 7036 |
| Corynebacterium | 9 | 0 | 971 | 4 | 283 | 972 | 380 | 1332 | 9 |
| Escherichia | 0 | 0 | 1 | 4 | 440 | 3 | 89 | 461 | 31 |
| Streptococcus | 6 | 0 | 512 | 18 | 1 | 0 | 396 | 2213 | 0 |
| Terrisporobacter | 0 | 0 | 931 | 1168 | 29 | 51 | 4 | 4 | 17 |
| Turicibacter | 2 | 0 | 491 | 363 | 2 | 142 | 7 | 4 | 50 |
| Sporobacter | 0 | 181 | 104 | 599 | 0 | 85 | 0 | 5 | 503 |
| Acinetobacter | 10 | 10 | 0 | 2 | 744 | 3 | 22 | 114 | 1165 |
| Enterococcus | 0 | 1 | 1 | 1 | 124 | 0 | 422 | 1735 | 6 |
| Oceanisphaera | 0 | 0 | 0 | 0 | 2437 | 0 | 0 | 4 | 0 |
| Ruminococcaceae_uc | 0 | 380 | 52 | 137 | 2 | 20 | 2 | 7 | 402 |
| Pseudomonas | 15 | 15 | 13 | 6 | 243 | 0 | 21 | 9 | 13 |
| Weissella | 0 | 0 | 0 | 0 | 0 | 0 | 0 | 0 | 0 |
| Prevotella | 0 | 0 | 43 | 1086 | 0 | 2 | 4 | 0 | 0 |
| PAC001207_g | 0 | 130 | 92 | 218 | 2 | 82 | 2 | 3 | 70 |
| Christensenellaceae_uc | 0 | 327 | 46 | 24 | 0 | 13 | 2 | 0 | 207 |
| Lachnospiraceae_uc | 0 | 448 | 21 | 152 | 2 | 4 | 0 | 2 | 18 |
| Peptostreptococcaceae_uc | 0 | 0 | 69 | 26 | 0 | 8 | 1 | 0 | 358 |
| Glutamicibacter | 0 | 0 | 0 | 0 | 708 | 0 | 36 | 172 | 31 |
| Treponema | 1 | 604 | 83 | 18 | 4 | 10 | 16 | 0 | 3 |
| PAC001437_g | 0 | 155 | 24 | 15 | 1 | 11 | 1 | 2 | 188 |
| Asaccharospora | 0 | 0 | 0 | 0 | 0 | 0 | 7 | 22 | 274 |
| Staphylococcus | 2 | 0 | 2 | 0 | 22 | 0 | 128 | 571 | 0 |
| HQ716072_g | 0 | 2 | 36 | 2 | 0 | 528 | 0 | 0 | 0 |
| Clostridiaceae_uc | 0 | 6 | 136 | 99 | 0 | 53 | 0 | 0 | 30 |
| AC160630_f_uc | 0 | 125 | 18 | 145 | 0 | 146 | 0 | 2 | 30 |
| PAC001115_g | 0 | 146 | 92 | 8 | 0 | 41 | 0 | 0 | 1 |
| Jeotgalicoccus | 1 | 0 | 16 | 1 | 171 | 0 | 48 | 227 | 2 |
| EU844681_g | 0 | 253 | 58 | 29 | 1 | 5 | 0 | 0 | 0 |
| AB494828_g | 0 | 340 | 17 | 33 | 0 | 11 | 1 | 0 | 0 |
| BF311_g | 0 | 414 | 0 | 0 | 0 | 0 | 0 | 0 | 0 |
| Enterobacter | 2 | 6 | 0 | 2 | 1 | 0 | 0 | 1 | 0 |
| Psychrobacter | 0 | 0 | 4 | 0 | 245 | 0 | 18 | 63 | 0 |
| Aerococcus | 0 | 0 | 0 | 0 | 3 | 0 | 55 | 177 | 0 |
| PAC002482_g | 0 | 0 | 1 | 0 | 0 | 61 | 0 | 0 | 54 |
| Kosakonia | 1 | 0 | 0 | 0 | 0 | 0 | 0 | 0 | 0 |
| Prevotellaceae_uc | 0 | 54 | 2 | 159 | 2 | 30 | 0 | 0 | 0 |
| 5-7N15_g | 0 | 0 | 0 | 0 | 0 | 0 | 0 | 0 | 159 |
| Lactobacillaceae_uc | 0 | 0 | 0 | 0 | 2 | 9 | 13 | 68 | 0 |
| Ruminococcus | 0 | 179 | 7 | 36 | 2 | 0 | 0 | 0 | 3 |
| Oscillibacter | 5 | 1 | 25 | 34 | 0 | 8 | 1 | 0 | 81 |
| Cellulosilyticum | 0 | 6 | 54 | 59 | 1 | 1 | 0 | 0 | 0 |
| Lysinibacillus | 2 | 0 | 0 | 0 | 267 | 1 | 1 | 1 | 0 |
| PAC000748_g | 0 | 35 | 2 | 3 | 0 | 7 | 0 | 0 | 114 |
| Alistipes | 0 | 0 | 0 | 0 | 1 | 0 | 0 | 2 | 179 |
| EU842575_g | 0 | 244 | 0 | 0 | 0 | 0 | 3 | 0 | 0 |
| PAC001168_g | 0 | 7 | 41 | 11 | 0 | 3 | 0 | 0 | 6 |

**Table S3: Metagenomic metabolic functional predictions derived from PICRUSt for manure samples**

| **Specific Metabolism in relation to genera** | **Cow1** | **Cow2** | **Cow3** | **Horse1** | **Horse2** | **Horse3** | **Pig1** | **Pig2** | **Pig3** | **Pathway** | **Metabolism Classification** | **Relevance to AD** |
| --- | --- | --- | --- | --- | --- | --- | --- | --- | --- | --- | --- | --- |
| Butanoate metabolism | 0.0 | 28.0 | 88.0 | 75.0 | 582.0 | 53.0 | 29.0 | 77.0 | 0.0 | map00650 | Carbohydrate metabolism | **Acidogenesis** |
| Glyoxylate and dicarboxylate metabolism | 0.0 | 22.0 | 52.0 | 16.0 | 95.0 | 24.0 | 16.0 | 44.0 | 0.0 | map00630 | Carbohydrate metabolism | **Acidogenesis** |
| Propanoate metabolism | 0.0 | 28.0 | 64.0 | 52.0 | 335.0 | 35.0 | 2 | 81.0 | 0.0 | map00640 | Carbohydrate metabolism | **Acidogenesis** |
| Starch and sucrose metabolism | 0.0 | 28.0 | 78.0 | 84.0 | 762.0 | 55.0 | 38.0 | 25.0 | 0.0 | map00500 | Carbohydrate metabolism | **Acidogenesis** |
| Fatty acid metabolism | 0.0 | 17.0 | 34.0 | 17.0 | 82.0 | 21.0 | 9.0 | 54.0 | 0.0 | map01212 | Fatty acid metabolism | **Acidogenesis** |
| Alanine, aspartate and glutamate metabolism | 0.0 | 37.0 | 101.0 | 68.0 | 53.0 | 54.0 | 47.0 | 58.0 | 0.0 | map00250 | Amino acid metabolism | **Acidogenesis and acetogenesis** |
| Amino acid metabolism | 0.0 | 9.0 | 25.0 | 14.0 | 114.0 | 22.0 | 1.0 | 19.0 | 0.0 | map00460 | Amino acid metabolism | **Acidogenesis and acetogenesis** |
| Arginine and proline metabolism | 0.0 | 47.0 | 136.0 | 53.0 | 389.0 | 76.0 | 51.0 | 78.0 | 0.0 | map00330 | Amino acid metabolism | **Acidogenesis and acetogenesis** |
| Glycine, serine and threonine metabolism | 0.0 | 37.0 | 93.0 | 51.0 | 389.0 | 41.0 | 33.0 | 6 | 0.0 | map00260 | Amino acid metabolism | **Acidogenesis and acetogenesis** |
| Histidine metabolism | 0.0 | 26.0 | 6 | 22.0 | 171.0 | 35.0 | 24.0 | 39.0 | 0.0 | map00340 | Amino acid metabolism | **Acidogenesis and acetogenesis** |
| Phenylalanine metabolism | 0.0 | 6.0 | 18.0 | 9.0 | 86.0 | 12.0 | 9.0 | 17.0 | 0.0 | map00360 | Amino acid metabolism | **Acidogenesis and acetogenesis** |
| Phenylalanine, tyrosine and tryptophan biosynthesis | 0.0 | 36.0 | 75.0 | 15.0 | 121.0 | 42.0 | 29.0 | 37.0 | 0.0 | map00400 | Amino acid metabolism | **Acidogenesis and acetogenesis** |
| Tyrosine metabolism | 0.0 | 12.0 | 37.0 | 57.0 | 462.0 | 27.0 | 15.0 | 34.0 | 0.0 | map00350 | Amino acid metabolism | **Acidogenesis and acetogenesis** |
| Cyanoamino acid metabolism | 0.0 | 8.0 | 23.0 | 11.0 | 97.0 | 13.0 | 12.0 | 1 | 0.0 | map00460 | Metabolism of other amino acids | **Acetogenesis** |
| D-Alanine metabolism | 0.0 | 5.0 | 13.0 | 17.0 | 139.0 | 8.0 | 5.0 | 12.0 | 0.0 | map00473 | Metabolism of other amino acids | **Acetogenesis** |
| D-Glutamine and D-glutamate metabolism | 0.0 | 6.0 | 16.0 | 12.0 | 89.0 | 8.0 | 7.0 | 9.0 | 0.0 | map00471 | Metabolism of other amino acids | **Acetogenesis** |
| Glutathione metabolism | 0.0 | 4.0 | 25.0 | 23.0 | 199.0 | 8.0 | 7.0 | 24.0 | 0.0 | map00480 | Metabolism of other amino acids | **Acetogenesis** |
| Selenocompound metabolism | 0.0 | 15.0 | 35.0 | 33.0 | 262.0 | 25.0 | 13.0 | 28.0 | 0.0 | map00450 | Metabolism of other amino acids | **Acetogenesis** |
| Taurine and hypotaurine metabolism | 0.0 | 2.0 | 11.0 | 12.0 | 112.0 | 4.0 | 4.0 | 8.0 | 0.0 | map00430 | Metabolism of other amino acids | **Acetogenesis** |
| Cysteine and methionine metabolism | 0.0 | 37.0 | 113.0 | 7 | 554.0 | 65.0 | 45.0 | 57.0 | 0.0 | map00270 | Amino acid metabolism | **Acetogenesis** |
| Nitrogen metabolism | 0.0 | 24.0 | 64.0 | 34.0 | 283.0 | 41.0 | 3 | 37.0 | 0.0 | map00910 | Energy metabolism | **Acetogenesis** |
| Biosynthesis of unsaturated fatty acids | 0.0 | 6.0 | 16.0 | 16.0 | 109.0 | 6.0 | 5.0 | 19.0 | 0.0 | map01040 | Lipid metabolism | **Acidogenesis** |
| Fatty acid biosynthesis | 0.0 | 23.0 | 53.0 | 66.0 | 49.0 | 29.0 | 2 | 45.0 | 0.0 | map00061 | Lipid metabolism | **Acidogenesis** |
| Glycerolipid metabolism | 0.0 | 16.0 | 34.0 | 44.0 | 343.0 | 23.0 | 11.0 | 29.0 | 0.0 | map00561 | Lipid metabolism | **Acidogenesis** |
| Glycerophospholipid metabolism | 0.0 | 21.0 | 54.0 | 5 | 369.0 | 33.0 | 2 | 44.0 | 0.0 | map00564 | Lipid metabolism | **Acidogenesis** |
| Linoleic acid metabolism | 0.0 | 4.0 | 8.0 | 26.0 | 199.0 | 2.0 | 3.0 | 6.0 | 0.0 | map00591 | Lipid metabolism | **Acidogenesis** |
| Primary bile acid biosynthesis | 0.0 | 1.0 | 0.0 | 7.0 | 51.0 | 0.0 | 0.0 | 2.0 | 0.0 | map00120 | Lipid metabolism | **Acidogenesis** |
| Secondary bile acid biosynthesis | 0.0 | 1.0 | 0.0 | 7.0 | 51.0 | 0.0 | 0.0 | 2.0 | 0.0 | map00121 | Lipid metabolism | **Acidogenesis** |
| Ethylbenzene degradation | 0.0 | 3.0 | 9.0 | 27.0 | 205.0 | 5.0 | 3.0 | 1 | 0.0 | map00642 | Xenobiotics biodegradation and metabolism | **Acidogenesis & acetogenesis** |
| Polycyclic aromatic hydrocarbon degradation | 0.0 | 3.0 | 9.0 | 17.0 | 144.0 | 4.0 | 4.0 | 9.0 | 0.0 | map00624 | Xenobiotics biodegradation and metabolism | **Acidogenesis & acetogenesis** |
| Aminobenzoate degradation | 0.0 | 8.0 | 18.0 | 43.0 | 322.0 | 1 | 4.0 | 31.0 | 0.0 | map00620 | Xenobiotics biodegradation and metabolism | **Acidogenesis (through acetyl CoA)** |
| Benzoate degradation | 0.0 | 13.0 | 36.0 | 5.0 | 393.0 | 22.0 | 11.0 | 44.0 | 0.0 | map00362 | Xenobiotics biodegradation and metabolism | **Acidogenesis (through acetyl CoA)** |
| Chloroalkane and chloroalkene degradation | 0.0 | 11.0 | 17.0 | 0.0 | 39.0 | 290.0 | 16.0 | 8.0 | 0.0 | map00625 | Xenobiotics biodegradation and metabolism | **Acidogenesis (through Formate & Pyruvate metabolism)** |
| Naphthalene degradation | 0.0 | 7.0 | 17.0 | 39.0 | 294.0 | 15.0 | 6.0 | 24.0 | 0.0 | map00626 | Xenobiotics biodegradation and metabolism | **Acidogenesis (through Succinyl-CoA and acetyl CoA)** |
| Pantothenate and CoA biosynthesis | 0.0 | 27.0 | 61.0 | 34.0 | 271.0 | 32.0 | 28.0 | 42.0 | 0.0 | map00770 | Metabolism of cofactors and vitamins | **Acidogenesis and acetogenesis (Synthesis and degradation of fatty acids)** |
| Riboflavin metabolism | 0.0 | 11.0 | 26.0 | 13.0 | 94.0 | 13.0 | 12.0 | 21.0 | 0.0 | map00740 | Metabolism of cofactors and vitamins | **Acidogenesis and acetogenesis (Synthesis and degradation of fatty acids)** |
| Phosphotransferase system (PTS) | 0.0 | 24.0 | 32.0 | 118.0 | 1033.0 | 85.0 | 12.0 | 31.0 | 0.0 | map02060 | Membrane transport | **Carbohydrate hydrolysis** |
| Fructose and mannose metabolism | 0.0 | 39.0 | 73.0 | 207.0 | 167 | 7 | 27.0 | 4 | 0.0 | map00051 | Carbohydrate metabolism | **Hydrolysis** |
| Galactose metabolism | 0.0 | 2 | 55.0 | 44.0 | 433.0 | 37.0 | 26.0 | 17.0 | 0.0 | map00052 | Carbohydrate metabolism | **Hydrolysis** |
| Peptidases | 0.0 | 69.0 | 181.0 | 13 | 1047.0 | 121.0 | 72.0 | 121.0 | 0.0 | 1002 | Genes and Proteins; Protein families: metabolism | **Hydrolysis** |
| Carbohydrate metabolism | 0.0 | 4.0 | 8.0 | 9.0 | 87.0 | 5.0 | 5.0 | 6.0 | 0.0 | 0 |  | **Hydrolysis** |
| Energy metabolism | 0.0 | 3 | 92.0 | 3 | 258.0 | 55.0 | 41.0 | 43.0 | 0.0 | 0 |  | **Hydrolysis** |
| Protein folding and associated processing | 0.0 | 19.0 | 55.0 | 34.0 | 274.0 | 27.0 | 22.0 | 38.0 | 0.0 | 0 |  | **Hydrolysis** |
| Toluene degradation | 0.0 | 1.0 | 6.0 | 9.0 | 88.0 | 1.0 | 1.0 | 1 | 0.0 | map00623 | Xenobiotics biodegradation and metabolism | **Hydrolysis (Carbon and energy source)** |
| Thiamine metabolism | 0.0 | 2 | 47.0 | 32.0 | 267.0 | 29.0 | 18.0 | 29.0 | 0.0 | map00730 | Metabolism of cofactors and vitamins | **Hydrolysis of carbohydrates (glucose)** |
| Pyruvate metabolism | 0.0 | 4 | 111.0 | 93.0 | 739.0 | 64.0 | 38.0 | 84.0 | 0.0 | map00620 | Carbohydrate metabolism | **Methanogenesis** |
| Methane metabolism | 0.0 | 49.0 | 107.0 | 81.0 | 636.0 | 65.0 | 44.0 | 63.0 | 0.0 | map00680 | Energy metabolism | **Methanogenesis** |
| Sulfur relay system | 0.0 | 1 | 31.0 | 16.0 | 129.0 | 19.0 | 9.0 | 24.0 | 0.0 | map04122 | Genetic Information Processing | **Methanogenesis** |
| Lysine biosynthesis | 0.0 | 31.0 | 83.0 | 56.0 | 434.0 | 48.0 | 32.0 | 47.0 | 0.0 | map00300 | Amino acid metabolism | N/A to AD |
| Valine, leucine and isoleucine biosynthesis | 0.0 | 3 | 72.0 | 29.0 | 234.0 | 37.0 | 31.0 | 44.0 | 0.0 | map00290 | Amino acid metabolism | N/A to AD |
| Valine, leucine and isoleucine degradation | 0.0 | 18.0 | 37.0 | 25.0 | 133.0 | 17.0 | 7.0 | 69.0 | 0.0 | map00280 | Amino acid metabolism | N/A to AD |
| Streptomycin biosynthesis | 0.0 | 1 | 27.0 | 28.0 | 223.0 | 14.0 | 12.0 | 9.0 | 0.0 | map00521 | Biosynthesis of other secondary metabolites | N/A to AD |
| Plant-pathogen interaction | 0.0 | 7.0 | 17.0 | 9.0 | 61.0 | 1 | 7.0 | 11.0 | 0.0 | map04626 | Environmental adaptation | N/A to AD |
| Glycosyltransferases | 0.0 | 9.0 | 22.0 | 4 | 364.0 | 13.0 | 1 | 21.0 | 0.0 | 1003 | Genes and Proteins; Protein families: metabolism | N/A to AD |
| Prenyltransferases | 0.0 | 13.0 | 28.0 | 3 | 233.0 | 14.0 | 12.0 | 25.0 | 0.0 | 1006 | Genes and Proteins; Protein families: metabolism | N/A to AD |
| Aminoacyl-tRNA biosynthesis | 0.0 | 49.0 | 111.0 | 106.0 | 815.0 | 59.0 | 51.0 | 88.0 | 0.0 | Map00970 | Genetic Information Processing; Translation | N/A to AD |
| Ribosome | 0.0 | 104.0 | 219.0 | 201.0 | 1597.0 | 107.0 | 107.0 | 159.0 | 0.0 | map03010 | Genetic Information Processing; Translation | N/A to AD |
| Ribosome Biogenesis | 0.0 | 63.0 | 139.0 | 122.0 | 926.0 | 67.0 | 57.0 | 101.0 | 0.0 | map03008 | Genetic Information Processing; Translation | N/A to AD |
| Staphylococcus aureus infection | 0.0 | 0.0 | 0.0 | 15.0 | 134.0 | 5.0 | 0.0 | 11.0 | 0.0 | map05150 | Human Diseases; Infectious disease: bacterial | N/A to AD |
| Bacterial secretion system | 0.0 | 22.0 | 53.0 | 37.0 | 272.0 | 22.0 | 2 | 49.0 | 0.0 | Map03070 | Membrane transport | N/A to AD |
| Limonene and pinene degradation | 0.0 | 7.0 | 16.0 | 37.0 | 262.0 | 9.0 | 3.0 | 31.0 | 0.0 | map00903 | Metabolism of terpenoids and polyketides | N/A to AD |
| Polyketide sugar unit biosynthesis | 0.0 | 6.0 | 16.0 | 14.0 | 111.0 | 8.0 | 8.0 | 4.0 | 0.0 | map00523 | Metabolism of terpenoids and polyketides | N/A to AD |
| Terpenoid backbone biosynthesis | 0.0 | 25.0 | 59.0 | 45.0 | 383.0 | 3 | 26.0 | 4 | 0.0 | map00900 | Metabolism of terpenoids and polyketides | N/A to AD |
| Pyrimidine metabolism | 0.0 | 77.0 | 191.0 | 156.0 | 1204.0 | 98.0 | 83.0 | 121.0 | 0.0 | map00240 | Nucleotide metabolism | N/A to AD |
| Two-component system | 0.0 | 87.0 | 18 | 73.0 | 503.0 | 104.0 | 7 | 171.0 | 0.0 | map02020 | Signal transduction | N/A to AD |
| Bacterial toxins | 0.0 | 4.0 | 12.0 | 7.0 | 64.0 | 8.0 | 4.0 | 9.0 | 0.0 | 2042 | Signaling and cellular processes | N/A to AD |
| Bisphenol degradation | 0.0 | 4.0 | 8.0 | 33.0 | 252.0 | 3.0 | 3.0 | 8.0 | 0.0 | map00363 | Xenobiotics biodegradation and metabolism | N/A to AD |
| Dioxin degradation | 0.0 | 1.0 | 4.0 | 1 | 85.0 | 7.0 | 2.0 | 6.0 | 0.0 | map00621 | Xenobiotics biodegradation and metabolism | N/A to AD |
| Butirosin and neomycin biosynthesis | 0.0 | 3.0 | 8.0 | 7.0 | 54.0 | 3.0 | 4.0 | 2.0 | 0.0 | 0 |  | N/A to AD |
| Drug metabolism - other enzymes | 0.0 | 13.0 | 39.0 | 3 | 229.0 | 17.0 | 16.0 | 17.0 | 0.0 | 0 |  | N/A to AD |
| Photosynthesis | 0.0 | 14.0 | 32.0 | 27.0 | 232.0 | 16.0 | 16.0 | 24.0 | 0.0 | map00195 | Energy metabolism | N/A to AD |
| Photosynthesis proteins | 0.0 | 15.0 | 32.0 | 27.0 | 232.0 | 16.0 | 16.0 | 26.0 | 0.0 | map00196 | Energy metabolism | N/A to AD |
| Purine metabolism | 0.0 | 93.0 | 215.0 | 223.0 | 1698.0 | 11 | 9 | 169.0 | 0.0 | map00230 | Nucleotide metabolism | N/A to AD (toxic in AD) |
| Citrate cycle (TCA cycle) | 0.0 | 17.0 | 46.0 | 38.0 | 282.0 | 3 | 11.0 | 5 | 0.0 | map00020 | Carbohydrate metabolism | Universal utility |
| Inositol phosphate metabolism | 0.0 | 5.0 | 7.0 | 1 | 67.0 | 3.0 | 1.0 | 14.0 | 0.0 | map00562 | Carbohydrate metabolism | Universal utility |
| Bacterial motility proteins | 0.0 | 78.0 | 172.0 | 16.0 | 49.0 | 129.0 | 63.0 | 99.0 | 0.0 | 2035 | Signaling and cellular processes | Universal utility |
| Cell motility and secretion | 0.0 | 6.0 | 12.0 | 8.0 | 62.0 | 8.0 | 6.0 | 13.0 | 0.0 | 0 |  | Universal utility |
| Inorganic ion transport and metabolism | 0.0 | 4.0 | 1 | 12.0 | 89.0 | 13.0 | 3.0 | 13.0 | 0.0 | 0 |  | Universal utility |
| Membrane and intracellular structural molecules | 0.0 | 11.0 | 34.0 | 21.0 | 171.0 | 15.0 | 17.0 | 34.0 | 0.0 | 0 |  | Universal utility |
| Metabolism of cofactors and vitamins | 0.0 | 5.0 | 16.0 | 8.0 | 64.0 | 12.0 | 6.0 | 13.0 | 0.0 | 0 |  | Universal utility |
| Pentose phosphate pathway | 0.0 | 35.0 | 75.0 | 71.0 | 604.0 | 46.0 | 3 | 42.0 | 0.0 | map00030 | Carbohydrate metabolism | Universal utility |
| Carbon fixation in photosynthetic organisms | 0.0 | 26.0 | 52.0 | 5 | 397.0 | 32.0 | 23.0 | 33.0 | 0.0 | map00710 | Energy metabolism | Universal utility |
| Carbon fixation pathways in prokaryotes | 0.0 | 34.0 | 105.0 | 61.0 | 451.0 | 58.0 | 35.0 | 76.0 | 0.0 | map00720 | Energy metabolism | Universal utility |
| Oxidative phosphorylation | 0.0 | 33.0 | 10 | 52.0 | 433.0 | 46.0 | 33.0 | 69.0 | 0.0 | map00190 | Energy metabolism | Universal utility |
| Bacterial chemotaxis | 0.0 | 43.0 | 94.0 | 15.0 | 49.0 | 54.0 | 4 | 59.0 | 0.0 | map02030 | Cellular Processes; Cell motility | Universal utility |
| Protein kinases | 0.0 | 18.0 | 22.0 | 21.0 | 151.0 | 15.0 | 1 | 33.0 | 0.0 | 1001 | Genes and Proteins; Protein families: metabolism | Universal utility |
| Chaperones and folding catalysts | 0.0 | 28.0 | 84.0 | 66.0 | 526.0 | 43.0 | 35.0 | 64.0 | 0.0 | 3110 | Genetic information processing | Universal utility |
| Chromosome | 0.0 | 66.0 | 159.0 | 14 | 1079.0 | 8 | 63.0 | 121.0 | 0.0 | 3036 | genetic information processing | Universal utility |
| DNA repair and recombination proteins | 0.0 | 121.0 | 29 | 245.0 | 1822.0 | 139.0 | 123.0 | 203.0 | 0.0 | 3400 | genetic information processing | Universal utility |
| DNA replication proteins | 0.0 | 49.0 | 135.0 | 101.0 | 784.0 | 61.0 | 56.0 | 87.0 | 0.0 | 3032 | Genetic information processing | Universal utility |
| Transcription factors | 0.0 | 9 | 194.0 | 125.0 | 1027.0 | 137.0 | 77.0 | 133.0 | 0.0 | 3000 | Genetic information processing | Universal utility |
| Transcription machinery | 0.0 | 44.0 | 12 | 51.0 | 363.0 | 6 | 52.0 | 61.0 | 0.0 | 3021 | Genetic information processing | Universal utility |
| Translation factors | 0.0 | 23.0 | 49.0 | 44.0 | 347.0 | 23.0 | 24.0 | 34.0 | 0.0 | 3000 | Genetic information processing | Universal utility |
| Protein export | 0.0 | 25.0 | 52.0 | 45.0 | 332.0 | 25.0 | 22.0 | 44.0 | 0.0 | map03060 | Genetic information processing | Universal utility |
| DNA replication | 0.0 | 27.0 | 66.0 | 57.0 | 439.0 | 32.0 | 28.0 | 52.0 | 0.0 | map03030 | Genetic information processing | Universal utility |
| Glycolysis / Gluconeogenesis | 0.0 | 43.0 | 92.0 | 11 | 882.0 | 72.0 | 39.0 | 8 | 0.0 | map00010 | Carbohydrate metabolism | Universal utility ( related to AD) |
| Amino sugar and nucleotide sugar metabolism | 0.0 | 49.0 | 117.0 | 157.0 | 1307.0 | 83.0 | 53.0 | 72.0 | 0.0 | Map00520 | Carbohydrate metabolism | Universal utility (Not directly related to AD) |
| Mismatch repair | 0.0 | 37.0 | 86.0 | 69.0 | 526.0 | 43.0 | 37.0 | 6 | 0.0 | map03430 | Genetic Information Processing; Replication and repair | Universal utility (Not directly related to AD) |
| Nucleotide excision repair | 0.0 | 18.0 | 4 | 36.0 | 259.0 | 21.0 | 18.0 | 27.0 | 0.0 | map03420 | Genetic Information Processing; Replication and repair | Universal utility (Not directly related to AD) |
| RNA polymerase | 0.0 | 8.0 | 16.0 | 19.0 | 144.0 | 8.0 | 7.0 | 14.0 | 0.0 | map03020 | Genetic Information Processing; Transcription | Universal utility (Not directly related to AD) |
| RNA transport | 0.0 | 6.0 | 1 | 15.0 | 113.0 | 8.0 | 4.0 | 1 | 0.0 | map03013 | Genetic Information Processing; Translation | Universal utility (Not directly related to AD) |
| Peptidoglycan biosynthesis | 0.0 | 3 | 85.0 | 66.0 | 533.0 | 48.0 | 37.0 | 57.0 | 0.0 | map00550 | Glycan biosynthesis and metabolism | Universal utility (Not directly related to AD) |
| ABC transporters | 0.0 | 204.0 | 363.0 | 198.0 | 1479.0 | 221.0 | 126.0 | 321.0 | 0.0 | Map02010 | Membrane transport | Universal utility (Not directly related to AD) |
| Secretion system | 0.0 | 56.0 | 125.0 | 8 | 634.0 | 83.0 | 44.0 | 104.0 | 0.0 | map03070 | Membrane transport | Universal utility (Not directly related to AD) |
| Folate biosynthesis | 0.0 | 15.0 | 38.0 | 21.0 | 159.0 | 24.0 | 17.0 | 32.0 | 0.0 | map00790 | Metabolism of cofactors and vitamins | Universal utility (Not directly related to AD) |
| Nicotinate and nicotinamide metabolism | 0.0 | 16.0 | 41.0 | 27.0 | 209.0 | 21.0 | 19.0 | 29.0 | 0.0 | map00760 | Metabolism of cofactors and vitamins | Universal utility (Not directly related to AD) |
| One carbon pool by folate | 0.0 | 23.0 | 61.0 | 41.0 | 324.0 | 35.0 | 28.0 | 36.0 | 0.0 | map00670 | Metabolism of cofactors and vitamins | Universal utility (Not directly related to AD) |
| Vitamin B6 metabolism | 0.0 | 9.0 | 2 | 9.0 | 67.0 | 8.0 | 1 | 12.0 | 0.0 | map00750 | Metabolism of cofactors and vitamins | Universal utility (Not directly related to AD) |
| Ion channels | 0.0 | 0.0 | 1.0 | 1 | 78.0 | 0.0 | 0.0 | 3.0 | 0.0 | map04750 | Sensory system | Universal utility (Not directly related to AD) |
| Phosphatidylinositol signaling system | 0.0 | 3.0 | 7.0 | 12.0 | 84.0 | 4.0 | 2.0 | 7.0 | 0.0 | map04070 | Signal transduction | Universal utility (Not directly related to AD) |
| Cytoskeleton proteins | 0.0 | 14.0 | 44.0 | 35.0 | 268.0 | 2 | 17.0 | 19.0 | 0.0 | 4812 | Signaling and cellular processes | Universal utility (Not directly related to AD) |
| Transporters | 0.0 | 363.0 | 668.0 | 502.0 | 4024.0 | 469.0 | 241.0 | 521.0 | 0.0 | 2000 | Signaling and cellular processes | Universal utility (Not directly related to AD) |
| Other ion-coupled transporters | 0.0 | 56.0 | 128.0 | 112.0 | 79 | 72.0 | 42.0 | 119.0 | 0.0 | 0 |  | Universal utility (Not directly related to AD) |
| Other transporters | 0.0 | 1 | 29.0 | 7.0 | 64.0 | 18.0 | 1 | 12.0 | 0.0 | 0 |  | Universal utility (Not directly related to AD) |
| Others | 0.0 | 4 | 84.0 | 152.0 | 1197.0 | 65.0 | 31.0 | 82.0 | 0.0 | 0 |  | Universal utility (Not directly related to AD) |
| Pores ion channels | 0.0 | 8.0 | 23.0 | 15.0 | 89.0 | 9.0 | 1 | 26.0 | 0.0 | 0 |  | Universal utility (Not directly related to AD) |
| Replication, recombination and repair proteins | 0.0 | 3 | 96.0 | 104.0 | 80 | 45.0 | 35.0 | 48.0 | 0.0 | 0 |  | Universal utility (Not directly related to AD) |
| Restriction enzyme | 0.0 | 4.0 | 12.0 | 22.0 | 182.0 | 9.0 | 6.0 | 11.0 | 0.0 | 0 |  | Universal utility (Not directly related to AD) |
| Signal transduction mechanisms | 0.0 | 22.0 | 46.0 | 47.0 | 369.0 | 33.0 | 16.0 | 39.0 | 0.0 | 0 |  | Universal utility (Not directly related to AD) |
| Translation proteins | 0.0 | 4 | 91.0 | 68.0 | 528.0 | 49.0 | 38.0 | 61.0 | 0.0 | 0 |  | Universal utility (Not directly related to AD) |
| Amino acid related enzymes | 0.0 | 58.0 | 141.0 | 106.0 | 845.0 | 76.0 | 63.0 | 97.0 | 0.0 | 01007 (BRITE) | Amino acid metabolism |  |
| Pentose and glucuronate interconversions | 0.0 | 2 | 44.0 | 27.0 | 246.0 | 28.0 | 22.0 | 13.0 | 0.0 | map00040 | Carbohydrate metabolism |  |
| Flagellar assembly | 0.0 | 32.0 | 76.0 | 4.0 | 27.0 | 63.0 | 23.0 | 35.0 | 0.0 | map02040 | Cellular Processes; Cell motility |  |
| Sulfur metabolism | 0.0 | 1 | 26.0 | 22.0 | 175.0 | 14.0 | 9.0 | 24.0 | 0.0 | map00920 | Energy metabolism | |
| Lipid biosynthesis proteins | 0.0 | 26.0 | 56.0 | 62.0 | 445.0 | 32.0 | 23.0 | 55.0 | 0.0 | 1004 | Genes and Proteins; Protein families: metabolism |  |
| Lipopolysaccharide biosynthesis proteins | 0.0 | 3.0 | 15.0 | 13.0 | 108.0 | 2.0 | 3.0 | 2 | 0.0 | 1005 | Genes and Proteins; Protein families: metabolism |  |
| Homologous recombination | 0.0 | 37.0 | 92.0 | 74.0 | 561.0 | 45.0 | 4 | 64.0 | 0.0 | map03440 | Genetic Information Processing; Replication and repair |  |
| Function unknown | 0.0 | 46.0 | 95.0 | 104.0 | 887.0 | 59.0 | 36.0 | 111.0 | 0.0 | 0 |  |  |
| General function prediction only | 0.0 | 156.0 | 355.0 | 231.0 | 182 | 201.0 | 136.0 | 258.0 | 0.0 | 0 |  |  |
|  |  |  |  |  |  |  |  |  |  |  |  |  |

**Note**: fonts in bold and placed in borders indicate area of focus and interpretation analysis for the study. Analysis was achieved following a two-step process, with the normalization of previously derived OTU table achieved with the script program command: **normalize_by_copy_number.py** performing the task function of dividing each OTU by the known/predicted 16S copy number abundance. The second step involved using the script command: **predict_metagenomes.py**, to predict functions for the metagenome (by multiplying each normalized OTU abundance by each predicted functional trait abundance to produce a table of functions (rows) by samples (columns).
